# Evaluation of the Postoperative Risk of Deep Tissue Injury to the Lower Extremities Following Surgery in the Lithotomy Position

**DOI:** 10.7759/cureus.57413

**Published:** 2024-04-01

**Authors:** Yohei Yukizawa, Emi Kamono, Shu Takagawa, Kunihito Hirotomi, Shota Higashihira, Hyonmin Choe, Yutaka Inaba, Naomi Kobayashi

**Affiliations:** 1 Orthopaedic Surgery, Yokohama City University Medical Center, Yokohama, JPN; 2 Orthopaedic Surgery, Yokohama City University, Yokohama, JPN

**Keywords:** laparoscopic surgery, lithotomy position, rhabdomyolysis, well-leg compartment syndrome (wlcs), compartment syndrome, deep tissue injury (dti)

## Abstract

Background

The aim of this study was to determine the incidence of deep tissue injury (DTI) and potential risk factors after surgery in the lithotomy position.

Methods

All patients who underwent surgery in the lithotomy position under general anesthesia at a single center between January 2017 and December 2021 were retrospectively evaluated. The medical records of these patients were reviewed, and patient demographic and clinical characteristics, surgical data, and occurrence of DTI were recorded.

Results

During the study period, 5146 patients, 2055 (39.9%) males and 3091 (60.1%) females, with a mean age of 57.3 ± 17.4 years, underwent surgery in the lithotomy position. Seven (0.14%) patients developed DTI on their calf following surgery. All presented with severe pain and swelling, requiring prolonged hospital stay. Multivariate analysis showed that male sex (odds ratio (OR): 11.43; 95% confidence interval (CI): 1.15-113.34, p = 0.037), higher BMI (OR: 1.32; 95% CI: 1.17-1.50, p = 0.0001), and longer operation time (OR: 1.01; 95% CI: 1.004-1.014, p = 0.0002) were independent risk factors for postoperative DTI. Optimal cut-off values for BMI and operation time were 23.5 kg/m^2^ (sensitivity = 100%; specificity = 64%) and 285 minutes (sensitivity = 100%; specificity = 90%), respectively.

Conclusion

Factors significantly associated with DTI include male sex, higher BMI, and prolonged operation time.

## Introduction

Rhabdomyolysis and skin pressure ulcers due to surgical position are potential complications in all surgical procedures. Deep tissue injury (DTI), consisting of injury to deep tissue with little or no damage to the skin, has been defined as a subtype of pressure ulcers [1]. Therefore, skeletal muscle and subcutaneous tissue pressure sores are now recognized as pressure ulcers. Because this definition is relatively recent, DTI is not yet widely recognized as a perioperative complication, and few studies, including case reports, have evaluated this complication.

During surgery in the lithotomy position, the patient is placed in a supine position, both lower extremities are raised and spread, and the lower legs are secured to a support. The lithotomy position has been utilized for diagnostic and therapeutic purposes mainly in patients undergoing urologic, gynecologic, and gastrointestinal surgery.

Placement of a patient in an anatomically and physiologically appropriate position is required to minimize injury to the nerves, muscles, and blood circulation. The lithotomy position can cause circulatory and neurologic complications in the lower extremities due to hip and knee flexion. Compromise may be localized, such as to the sacral region [2,3], but it may also be widespread, involving the entire lower leg, and may result in peroneal nerve palsy and compartment syndrome. Compartment syndrome is a complication that requires prompt treatment, such as surgical decompression, before progression and is likely to require restriction of activities for several weeks afterward [4-7]. In addition, patients may experience perioperative complications unrelated to the underlying disease.

The number of robotic-assisted laparoscopic surgical procedures is increasing worldwide, including in our institution, making the prevention of perioperative complications due to lithotomy a matter of concern for orthopedic surgeons. It is therefore important to determine the incidence of and risk factors for lower extremity DTI in patients undergoing surgery in the lithotomy position. The aim of this study was therefore to retrospectively determine the incidence of DTI in patients undergoing surgery in the lithotomy position and to identify the risk factors associated with DTI in these patients.

## Materials and methods

This cross-sectional study included all patients aged >16 years at our institution who underwent surgery in the lithotomy position under general or spinal anesthesia between January 1, 2017, and December 31, 2022. This investigation complied with the Declaration of Helsinki and was approved by our institutional review board. Informed consent was obtained from all the study participants by opt-out method on the website. Factors recorded included patient demographic characteristics, such as gender, age, height, weight, and body mass index (BMI), primary disease, surgical site, presence of malignancy, operative technique, laparoscopic use, operation time, and blood loss.

To identify patients with DTI complications, the medical records of eligible patients were searched for the phrases "compartment," "well-leg compartment syndrome (WLCS)," "rhabdomyolysis," "leg pain," "pain on the buttocks," "calf pain," and "swelling of the buttocks or leg," or their synonyms. Patients with serum creatinine kinase (CK) concentrations ≥1000 IU/L within seven days after surgery and those with a history of postoperative consultation with an orthopedic surgeon were also identified. The perioperative medical records of all selected patients were subsequently reviewed in detail by two orthopedic surgeons to identify those with DTI. The diagnostic criteria included severe postoperative pain and swelling in any part of the leg from the temple to the lower extremity for at least two days not caused by deep vein thrombosis or infection.

The sample size was set as follows: a postoperative incidence rate of WLCS was reported as 0.2%, and the risk factor of prolonged surgery was also reported [8]. The sample size for the logistic analysis was calculated to require about 5000 surgical cases overall. Univariate and multivariate analyses were performed to identify factors associated with the development of postoperative DTI. Factors evaluated included gender, age, height, weight, BMI, operation time, blood loss, laparoscopic (yes vs. no), and malignancy (yes vs. no). All statistical analyses were performed using JMP software (SAS Institute Inc., Cary, NC), with p < 0.05 considered statistically significant.

## Results

**Figure 1:**
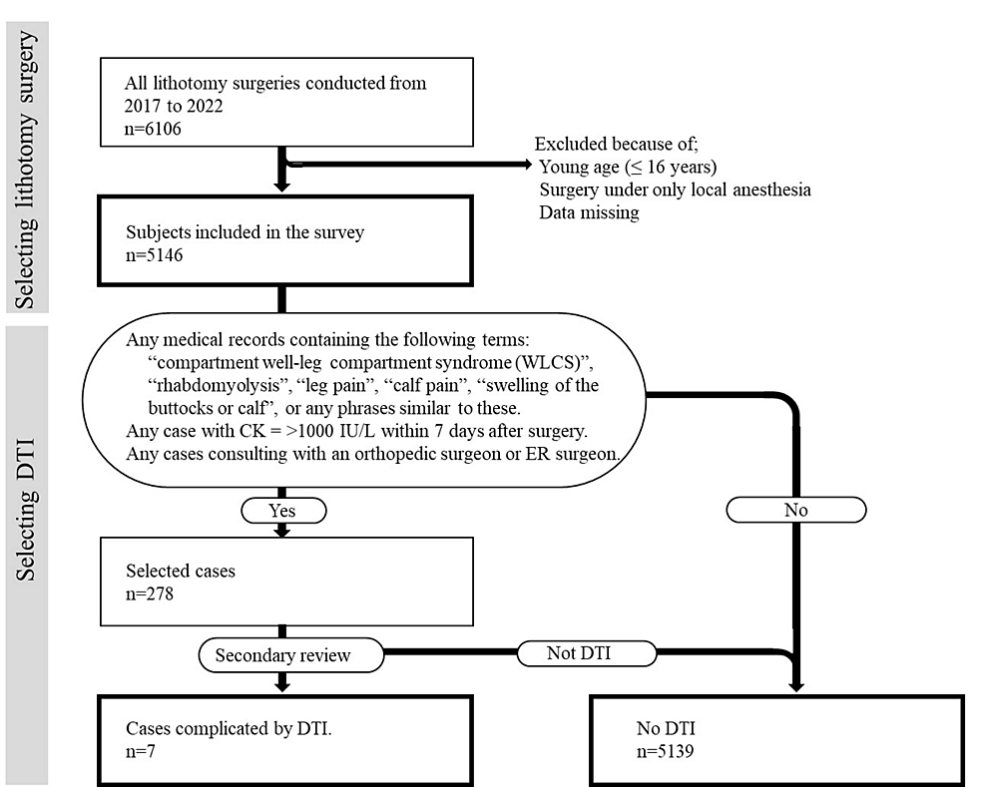
Flowchart of patients who underwent surgery in the lithotomy position and did and did not develop postoperative DTI DTI: deep tissue injury; CK: creatinine kinase.

Figure 1 shows the flowchart of patient inclusion and exclusion criteria. During the study period, a total of 5311 patients underwent surgery in the lithotomy position at our institution. After excluding patients with missing data and those aged <16 years, 5146 patients, consisting of 2055 (39.9%) males and 3091 (60.1%) females, with a mean age of 57.3 ± 17.4 years, were included (Table 1). Of these patients, 2212 (43.0%) underwent digestive surgery, 1842 (35.8%) underwent gynecologic surgery, 928 (18.0%) underwent urologic surgery, 106 (2.1%) underwent obstetric surgery, and 58 (1.1%) underwent other types of surgery. Moreover, 2861 (55.6%) patients underwent laparoscopic surgery and 2839 (55.2%) underwent surgery for a malignant tumor.

**Table 1:** Demographic and clinical characteristics of the study population

Total number, n	5146
Gender	
Male, n (%)	2055 (39.9)
Female, n (%)	3091 (60.1)
Age at surgery, year, mean ± SD	57.3 ± 17.4
Department in charge	
Gastrointestinal surgery, n (%)	2212 (43.0)
Gynecology, n (%)	1842 (35.8)
Urology, n (%)	928 (18.0)
Obstetrics, n (%)	106 (2.1)
Other, n (%)	58 (1.1)
Surgery for a malignant tumor, n (%)	2839 (55.2)
Use of laparoscopy, n (%)	2861 (55.6)
Operation time, minutes, mean ± SD	148 ± 112
Blood loss, mL, mean ± SD	129 ± 380

Preoperatively, patients were placed in the lithotomy position by setting the lower extremities on the leg holders and placing an intermittent pneumatic compression device on the lower legs. Intraoperatively, patients were covered with drapes, but no decompression procedures of the lower extremities were attempted.

Of the 5146 patients, seven (0.14%) experienced postoperative DTI complications. These seven patients included six males and one female, with a mean age of 48.0 ± 3.3 years and a mean BMI of 29.0 ± 4.4 kg/m^2^. All seven patients underwent laparoscopic surgery, with a mean operative time of 361 ± 98 minutes and a mean intraoperative blood loss of 245 ± 233 mL. Serum concentrations of CK were measured immediately after surgery in three patients, with these concentrations found to be 29807 U/L, 48158 U/L, and 5083 U/L, respectively. Compartment pressure was measured in two of the seven patients and was found to be 31 mmHg and 43 mmHg, respectively. None of the seven patients developed progressive circulatory or neurological deficits due to leg swelling, and all recovered spontaneously without surgical procedures for decompression. However, postoperative weaning was delayed due to gait disturbance caused by pain, prolonging hospital stay by an average of six days.

**Figure 2:**
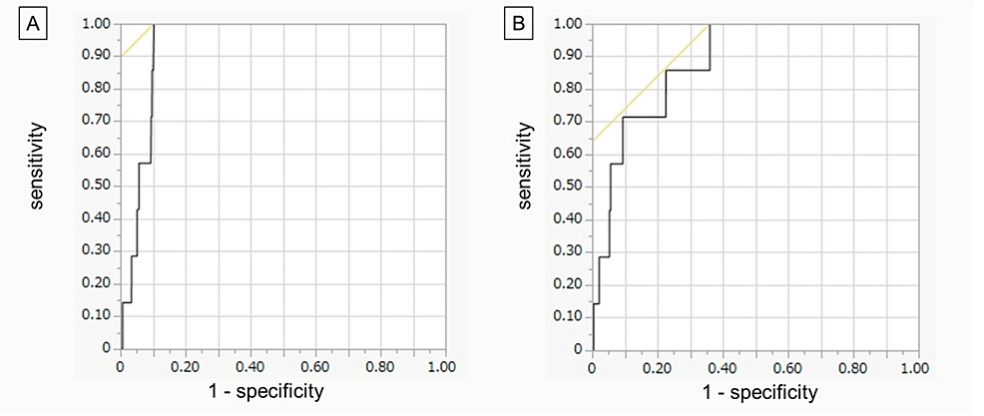
Receiver operating characteristic (ROC) analysis of body mass index (BMI) and operation time The areas under the curve were 0.883 for BMI (A) and 0.936 for operation time (B), both of which were statistically significant (p < 0.0001 each). The cut-off values with the highest values of “sensitivity-(1-specificity)” in each ROC curve were BMI = 23.51 kg/m^2^ (sensitivity = 100%; specificity = 64%) and operation time = 285 minutes (sensitivity = 100%; specificity = 90%).

Univariate analysis showed that male sex, higher BMI, longer operation time, laparoscopic surgery, and surgery for malignancy were significantly associated with DTI complications (p < 0.01 each, Table 2). Multivariate logistic regression analysis was performed to determine whether these factors were independently associated with the risk of DTI. However, "laparoscopy" and "malignancy," which had frequencies of 0 in the DTI-free group, were excluded because they could not fit into the analytical model for statistical reasons. Multivariate logistic regression analysis showed that the chi-square statistic for the overall analytical model was 33.73 (p < 0.0001), indicating that the model was significant for the analysis. Male sex (odds ratio (OR): 11.43; 95% confidence interval (CI): 1.15-113.34, p = 0.037), higher BMI (OR: 1.32; 95% CI: 1.17-1.50, p = 0.0001), and longer operation time (OR: 1.01; 95% CI: 1.004-1.014, p = 0.0002) were all found to be significant factors affecting DTI complications (Table 3). Receiver operating characteristic (ROC) curves plotted for BMI and operation time yielded areas under the curve of 0.883 and 0.936, respectively, both of which were statistically significant (p < 0.0001) (Figure 2). The cut-off values with the highest values of sensitivity-(1-specificity) on each ROC curve were BMI = 23.51 kg/m2 (sensitivity = 100%; specificity = 64%) and operation time = 285 minutes (sensitivity = 100%; specificity = 90%).

**Table 2:** Univariate analysis of factors in patients with and without postoperative deep tissue injury * Fisher's exact test. ** Univariate logistic regression analysis. DTI: deep tissue injury.

	DTI		p-value
	No (n = 5139)	Yes (n = 7)	
Gender			
Male, n (%)	2049 (39.8)	6 (0.12)	0.0019^*^
Female, n (%)	3090 (60.0)	1 (0.08)	
Age at surgery, years, mean ± SD	57.3 ± 17.4	48.0 ± 3.3	0.165**
Body mass index, kg/m^2^, mean ± SD	22.6 ± 3.9	29.0 ± 4.4	<0.0001**
Surgery for a malignant tumor, n (%)	2832 (55.0)	7 (0.14)	0.019*
Use of laparoscopy, n (%)	2854 (55.5)	7 (0.14)	0.020*
Operation time, minutes, mean ± SD	148 ± 112	361 ± 98	<0.0001**
Blood loss, mL, mean ± SD	129 ± 380	245 ± 233	0.440

**Table 3:** Multivariate logistic regression analysis of factors associated with deep tissue injury

	Odds ratio	Upper 95% CI	Lower 95% CI	p-value
Male	11.43	1.152	113.342	0.0374
Body mass index	1.321	1.171	1.501	0.0001
Operation time	1.009	1.004	1.014	0.0002

A subanalysis was performed on patients who underwent laparoscopic surgery in the lithotomy position. Univariate analyses showed that younger age, male sex, higher BMI, longer operation time, and surgery for malignancy were significantly associated with DTI complications (p < 0.05 each; Table 4). Multivariate logistic regression analysis showed that only younger age (OR: 0.921; 95% CI: 0.869-0.976, p = 0.018), higher BMI (OR: 1.268; 95% CI: 1.109-1.450, p = 0.0061), and longer operation time (OR: 2.157; 95% CI: 1.361-3.420, p = 0.0017) were independent factors significantly associated with DTI complications.

**Table 4:** Results of multivariate logistic regression analysis in patients who underwent laparoscopic surgery

	Odds ratio	Upper 95% CI	Lower 95% CI	p-value
Younger age	0.921	0.869	0.976	0.018
Body mass index	1.268	1.109	1.450	0.0061
Operation time	2.157	1.361	3.420	0.0017

Another subanalysis was also performed on patients who underwent surgery for malignant tumors in the lithotomy position. Univariate analyses showed that younger age, higher BMI, longer operation time, and laparoscopic surgery were significantly associated with DTI complications (p < 0.01 each; Table 5). Multivariate logistic regression analysis showed that younger age (OR: 0.890; 95% CI: 0.825-0.959, p = 0.0010), higher BMI (OR: 1.256; 95% CI: 1.101-1.433, p = 0.0023), and longer operation time (OR: 1.799; 95% CI: 1.232-2.628, p = 0.0002) were independent factors significantly associated with DTI complications.

**Table 5:** Results of multivariate logistic regression analysis in patients who underwent surgery for malignancy

	Odds ratio	Upper 95% CI	Lower 95% CI	p-value
Younger age	0.890	0.825	0.959	0.0010
Body mass index	1.256	1.101	1.433	0.0023
Operation time	1.799	1.232	2.628	0.0002

## Discussion

The present study assessed the incidence of DTI in patients who underwent surgery in the lithotomy position as well as determining the factors associated with DTI in this population. Evaluation of pressure in the lithotomy position showed that pressure was significantly higher on the proximal calf than on other parts of the body, with pressure exceeding 50 mmHg being associated with obesity and elevation of the lower limb above 60 degrees [9]. Although the present study also found that high BMI was a risk factor for DTI, the calculated cutoff values were not limited to obese individuals, with non-thin patients considered to be at uniform risk of developing DTI.

Another risk factor, the length of time spent in the lithotomy position, was also associated with the occurrence of DTI, with a cutoff value of 285 minutes. Several other studies have reported that prolonged surgery, especially longer than four hours, is a risk factor for DTI [10-13]. Intraoperative lower extremity compartment pressures steadily increase over time, exceeding perfusion pressures after an average of five hours of continuous positioning [14]. One of the factors associated with the pathogenesis of DTI is placing the lower extremities at a higher position than the heart [15]. This placement reduces perfusion pressure, as well as affects external pressure, thereby creating an environment conducive to anaerobic metabolism within the tissues [15]. This, in turn, increases the permeability of peripheral blood vessels and causes inflammatory tissue damage in the third space. Normally, this change is reversed by repositioning the lower extremities, but, in some patients, it may trigger reperfusion, which in turn induces secondary tissue damage, resulting in DTI [15,16].

In the present study, male sex was a factor significantly associated with postoperative DTI. In general, men are larger and have more muscle mass than women. Muscle mass and circumference of the lower extremities have been associated with the occurrence of postoperative DTI [17,18], findings consistent with the results of the present study.

All patients who developed DTI in this study had undergone laparoscopic procedures to remove malignant tumors. Therefore, these two factors could not be statistically analyzed as being associated with the occurrence of DTI. However, the mean operation time of laparoscopic surgery for malignant tumors was significantly longer than the mean operation times of other procedures (Tables 4, 5), suggesting that laparoscopic surgery and malignancy were likely confounding factors for the operation time. Moreover, laparoscopic surgery for malignant tumors required more frequent intraoperative rotations of the operating table, which may have caused pressure imbalance in the lower legs.

Large epidemiological studies have shown that DTIs are rare in patients undergoing lithotomy. For example, the incidence of DTI in the UK was found to be one per 3500 [11]. An additional survey, however, found that many DTIs are unreported [19]. The present study found that the incidence of DTI was one per 735, which is higher than previously reported.

Several interventions to prevent DTI during surgery have been reported to reduce its occurrence [20]. In that study, surgery was performed in the open leg position with minimal or no elevation of the lower extremities, if technically feasible, with the patient's body leveled every three hours. Although problems may arise because the lower extremities are completely covered by drapes, it is important that such measures be planned and shared with the operating room staff prior to starting surgery [21].

Despite DTI being a familiar perioperative complication of lithotomy, it has not been well documented in clinical investigations. DTI is a relatively rare perioperative complication of various types of surgery and can present with various indications, including compartment syndrome, rhabdomyolysis, and WLCS. General surveys are lacking because this complication has been assessed only in small studies and case reports of patients presenting with various indications. Utilizing a clinical database, as in this study, may enable a determination of the incidence of DTI and an analysis of factors associated with this complication.

The present study had several limitations. First, this study analyzed patients who underwent surgery at a single center. The results of this study may therefore not reflect the risks of DTI in patients undergoing surgery in the lithotomy position at other centers or in multiple centers. Since DTI is a rare complication, statistical analysis with an even larger sample size is needed. Second, the criteria used to identify patients with DTI may not have identified all patients with this complication. In particular, minor DTIs with mild pain may not have been recorded in patients’ medical records. Third, the duration period of lithotomy position might vary because this study included the types of surgeries. Also, head-down or left-right rotation might be set, though the medical records of these patients were not accurately recorded. Increased pressure on the lower leg has been reported in patients placed in the Trendelenburg position [18], indicating a need for further detailed investigations.

## Conclusions

The incidence of DTI after lithotomy surgery in this study was 0.14%. Although a rare surgical complication, DTI of the lower extremities is associated with severe pain and gait disturbance, leading to prolonged hospitalization. Preventive measures are essential, especially for high-risk patients. Male sex, high BMI, and long operation time were found to be risk factors for the occurrence of postoperative DTI. Patients should be monitored carefully, especially if they have a BMI > 25 kg/m^2^ and the operation time is ≥ four hours.
